# BrainWave: A Matlab Toolbox for Beamformer Source Analysis of MEG Data

**DOI:** 10.3389/fnins.2018.00587

**Published:** 2018-08-22

**Authors:** Cecilia Jobst, Paul Ferrari, Silvia Isabella, Douglas Cheyne

**Affiliations:** ^1^Program in Neurosciences and Mental Health, The Hospital for Sick Children, Toronto, ON, Canada; ^2^MEG Laboratory, Dell Children’s Medical Centre of Central Texas, Austin, TX, United States; ^3^Department of Medical Imaging, University of Toronto, Toronto, ON, Canada

**Keywords:** Matlab toolbox, source analysis, magnetoencephalography, beamforming, group analysis, response inhibition

## Abstract

*BrainWave* is an easy-to-use Matlab toolbox for the analysis of magnetoencephalography data. It provides a graphical user interface for performing minimum-variance beamforming analysis with rapid and interactive visualization of evoked and induced brain activity. This article provides an overview of the main features of *BrainWave* with a step-by-step demonstration of how to proceed from raw experimental data to group source images and time series analyses. This includes data selection and pre-processing, magnetic resonance image co-registration and normalization procedures, and the generation of volumetric (whole-brain) or cortical surface based source images, and corresponding source time series as virtual sensor waveforms and their time-frequency representations. We illustrate these steps using example data from a recently published study on response inhibition (Isabella et al., 2015) using the sustained attention to response task paradigm in 12 healthy adult participants. In this task participants were required to press a button with their right index finger to a rapidly presented series of numerical digits and withhold their response to an infrequently presented target digit. This paradigm elicited movement-locked brain responses, as well as task-related modulation of brain rhythmic activity in different frequency bands (e.g., theta, beta, and gamma), and is used to illustrate two different types of source reconstruction implemented in the *BrainWave* toolbox: (1) event-related beamforming of averaged brain responses and (2) beamformer analysis of modulation of rhythmic brain activity using the synthetic aperture magnetometry algorithm. We also demonstrate the ability to generate group contrast images between different response types, using the example of frontal theta activation patterns during error responses (failure to withhold on target trials). *BrainWave* is free academic software available for download at http://cheynelab.utoronto.ca/brainwave along with supporting software and documentation. The development of the *BrainWave* toolbox was supported by grants from the Canadian Institutes of Health Research, the National Research and Engineering Research Council of Canada, and the Ontario Brain Institute.

## Introduction

Magnetoencephalography (MEG) involves the measurement of the magnetic fields generated by the electrical currents that flow in activated neuronal circuits of the brain (Hämäläinen et al., 1993; Cheyne and Papanicolaou, 2013). A major advantage of MEG over other brain imaging methods is the ability to estimate location, strength, and time courses of these neuronal currents by using inverse modeling of electrical brain sources and co-registering such sources to a participant’s own anatomical magnetic resonance image (MRI) – a technique referred to as magnetic source imaging (Cheyne and Papanicolaou, 2013). In addition to providing a neuroanatomical interpretation to the estimated neural activity, the use of source-space analysis of brain activity also overcomes the problem of the superposition, or ‘mixing,’ of activity from multiple neural sources (and other magnetic sources such as muscle activity) at the sensors outside of the head, thereby increasing the ability to separate and identify the underlying neural generators (Baillet, 2017). This requires a solution to the so-called inverse problem, which states there is no unique configuration of sources for an externally measured field pattern (Helmholtz, 1853). For simple source configurations, standard parametric models (e.g., equivalent current dipoles) can be fitted to the data. However, this becomes a highly underdetermined mathematical problem for complex and distributed configurations of multiple sources, such as those associated with higher cognitive function or sources embedded within noise from magnetic artifacts, requiring more advanced source estimation methods.

A variety of methods have been applied to the MEG source estimation problem (Hämäläinen et al., 1993; Darvas et al., 2004; Hillebrand and Barnes, 2005; Cheyne and Papanicolaou, 2013; Baillet, 2017). An increasingly popular approach over the last decade is the signal processing technique known as *minimum-variance beamforming*, a spatial filtering method that utilizes the coincident detection of signals at multiple sensors to selectively enhance or suppress signals arising from different spatial locations, allowing for the simultaneous separation of multiple brain and external noise sources. Beamforming thus has an advantage over other inverse methods in that it provides a means for the extraction (un-mixing) of multiple sources of neural activity embedded within noisy data, often without the need for artifact removal or denoising of the raw data (Cheyne et al., 2006). Various beamforming methods have been introduced for source reconstruction of brain activity using MEG data (Van Veen et al., 1997; Robinson and Vrba, 1999; Gross et al., 2001; Sekihara et al., 2001; Cheyne et al., 2007). One popular implementation, termed synthetic aperture magnetometry (*SAM*), uses a minimum-variance beamformer algorithm to estimate whole-brain images of source power with user-defined frequency ranges and time windows. It also introduced metrics for estimating changes in source power between time windows or experimental conditions (Robinson and Vrba, 1999). The beamforming method can also be applied to the modeling of averaged phase-locked event-related brain activity (i.e., evoked responses), which we term “event-related” beamforming or *ERB* (Bardouille et al., 2004; Cheyne et al., 2006, 2007). The theoretical and computational bases of SAM and ERB are described in detail in numerous previous publications (Robinson and Vrba, 1999; Hillebrand and Barnes, 2005; Sekihara et al., 2005; Cheyne et al., 2006) as well as in the *BrainWave* documentation and are not repeated here. Both methods have been implemented in *BrainWave* to provide a common preprocessing and visualization platform, and to allow for the analysis and direct comparison of both evoked and induced brain activity within one toolbox.

At the time of this publication, *BrainWave* version 3.5 was the latest available software release. Using data collected from our previously published study on response inhibition (Isabella et al., 2015) we will demonstrate a standard workflow in bringing raw MEG data to a publishable group analyses (a copy of these data have been provided for download here https://figshare.com/s/2e1c6559cadae29429bc). In doing so, we will highlight the use of the two beamformer algorithms available in this toolbox, illustrating the localization of transient evoked motor responses (*using ERB beamformer*), and task-related narrow-band oscillatory modulations of induced brain rhythmic activity (*using the SAM beamformer*). In addition, the high error rates in the example data allow us to demonstrate the identification of oscillatory changes associated with error processing using a built-in module for computing between-condition contrast images, along with time-frequency analysis of source waveforms from group averaged source activity. We also illustrate the ability to create source images volumetrically (using predefined whole-brain volumes with variable spatial resolution), which can be aligned to standard template (MNI) brain space with automatic labeling using brain atlases. Alternatively, source activity can be computed on extracted cortical surfaces (Lin et al., 2006) imported from either the *Freesurfer* or *CIVET* software packages (example surfaces are provided with the demonstration data, with the additional steps described at the end of the tutorial).

The following sections will provide a basic step-by-step workflow example to bring a typical raw MEG dataset to the group plots of source images and time series analyses. It should be noted that most, but not all features will be demonstrated, and that additional details and suggestions for parameter selection can be found in the documentation provided with the *BrainWave* toolbox, both available at http://cheynelab.utoronto.ca/brainwave.

## Demonstration Datasets

The example MEG datasets were collected on a 151-channel CTF system (1200 Samples/s) within a Vacuumschmelze magnetically shielded room (Ak3b) with continuous head localization (CHL) enabled. Data was acquired in 12 healthy adults (five females, range: 21–35 years, all right-hand dominant) with informed consent in accordance with the Declaration of Helsinki from all participants as per The Hospital for Sick Children Research Ethics Board policies.

Participants were recorded in the seated position, and visual stimuli presented on a back-projection visual display. In the original study, two separate tasks were performed in a counterbalanced order across participants: (1) a standard “Go/No-go” version of the sustained attention to response task (SART) (Robertson et al., 1997) that involved withholding button press responses to an infrequently presented target stimulus within a rapidly presented stream of “Go” stimuli, and (2) a “Go/Switch” variation of the SART task that was identical to the Go/No-go task except that participants were instructed to switch response hands, rather than withhold responses, to target trials (Cheyne et al., 2012).

Each experimental trial in the withhold task presented a single numerical digit (the numbers ‘1’ to ‘9’) for 400 ms duration, immediately followed by a stimulus mask (the letter ‘X’) that remained for a variable duration inter-stimulus interval of 1800–2200 ms. Participants were instructed to press a button with their dominant (right) index finger when a number appeared, as quickly and accurately as possible. When the target stimulus appeared (the number ‘3,’ presented at a 20% probability rate), participants were instead instructed to withhold their response. Further details of the experimental setup and design can be found in Isabella et al. (2015).

To reduce the file size of the example data, we include only the Go/No-go (withhold) data for demonstration purposes and have downsampled the datasets to 600 Samples per second using *BrainWave’s* downsampling feature (not described here). In addition, event markers have been sorted by the experimenter to identify trial types and reduce the very large number of correct default (‘Go’) trials to aid in computation time on some computers. No other preprocessing has been applied to the MEG data. However, some preprocessing was required for the use of participant MRIs. Due to research ethics requirements on data sharing, MRIs have been de-identified offline using a de-facing tool (Bischoff-Grethe et al., 2007) that had rendered some *BrainWave* features unusable for this demonstration. For example, *BrainWave* is designed to work with *FMRIB Software Library (FSL*, version 5 or newer*)* (Smith, 2002; Jenkinson et al., 2012) *Brain Extraction Tool (BET2*) to generate segmented MRI surfaces for spherical model fitting, which requires access to facial features of the MRI to accurately model the brain’s surface. Because of this, the placement of MEG head localization coil positions located between the eyes, and adjacent to each ear (required for the co-registration or alignment of MEG to MRI data) is not possible in defaced images. For this reason we provide fully pre-processed MRI files in the sample data, with preselected co-registration information (i.e., head localization coils), as well as pre-computed FSL surfaces and a high-resolution pial cortical surface mesh using the CIVET (Ad-Dab’bagh et al., 2005) software package. While the steps to create FSL or CIVET surfaces will not be described in full, Section “Preparing MRI Data” will instruct the use of the pre-computed components for the generation of a spherical head model calculation (from FSL surfaces), and the use of high-resolution (CIVET) cortical surfaces for surface-constraint beamforming and 3D rendering of individual subject or template brain surfaces using *BrainWave*.

For demonstration purposes, we describe below the steps necessary to re-analyze these data using the most recent version of the *BrainWave* toolbox in a subset of the responses described above (i.e., **correct default** – the correct button press response to a non-target stimulus – and **error withhold** – the incorrect button press response to target trials or failure to ‘withhold’). This tutorial will illustrate how to analyze movement-locked evoked responses using the ERB algorithm and modulation of narrow-band oscillatory analysis of induced brain activity using the SAM beamformer algorithm, including frontal theta band (4–8 Hz) oscillations elicited on errors trials, and modulation of sensorimotor beta band (15–30 Hz) activity preceding and following motor responses. Source activity will be aligned to anatomical locations after co-registering (aligning) the MEG sensor data with the participant’s own anatomical T1 MRI images (Siemens 3T, MPRAGE) collected on the same day.

## *BrainWave* Software

### Compatible Data Formats and System Requirements

#### MEG Data Formats

*BrainWave* uses the native CTF MEG4 data format for reading and writing MEG data files, which are directories containing all files for a single data collection ending with a *.ds* extension. This allows for inter-operability of any MEG dataset with the CTF MEG4 tools or *BrainWave*, as well as any other software packages compatible with the CTF format. Other compatible MEG manufacturers, *Elekta/Neuromag*^1^ and *KIT/Yokogawa*^2^ may also be imported into *BrainWave* using the *Import MEG* feature and are converted into a CTF dataset format. Using a common dataset format allows for a standardized cross-platform approach for MEG source analysis. Once converted, subsequent processing and analyses will remain identical to CTF datasets. Importantly, these conversion programs can be run from within the *BrainWave* toolbox, in which case event markers (e.g., Elekta/Neuromag stimulus channel events) will be automatically converted to CTF MarkerFile format events. While the examples shown here involve the use of data recorded from a CTF MEG system, *BrainWave* has been tested with data from both Elekta and KIT MEG systems. For this purpose, we include options for selecting sensor types (magnetometers or gradiometers) as well as covariance matrix regularization for data that has been transformed using software-denoising schemes (e.g., signal space separation or ICA). These options are discussed in more detail in corresponding sections.

#### MRI Data Formats

Anatomical (T1) MRI data can be imported from Neuroimaging Informatics Technology Initiative (NIfTI,.nii), CTF (.mri) and raw DICOM3 (e.g., .ima or .dcm) formats, and are reformatted and saved into a standardized (RAS) NIfTI format within *BrainWave*. Tri-linear interpolation will automatically convert non-isotropic MRI data to the smallest equal voxel dimension to fit within a 256 × 256 × 256 voxel volume.

#### System Requirements

##### Hardware

*BrainWave* integrates high-level algorithms for beamformer source analysis with minimal user set up to bring raw data to interpretable results. Integrated compiled *C*-mex functions written in C++ offer efficient handling of MEG data, and uses multi-threaded libraries for rapid computation of beamformer images – ideally suited for multi-core processors with a minimum of 4 GB RAM. Mex-files are currently provided for Linux (64-bit), Windows (64-bit, Windows 10 recommended), and Mac OS (64-bit, version 10.6 or newer).

##### Software

No custom Matlab toolboxes are required to run *BrainWave*. For spatial normalization and group imaging, *BrainWave* can automatically warp source images to MNI template space, which requires the installation of *Statistical Parametric Mapping (SPM8 and SPM12 currently supported)* (Friston et al., 2007), and includes optional scaling to Talairach coordinates with an integrated brain atlas^3^ for automatic labeling of brain regions.

*BrainWave* is designed to work with two additional software packages for advanced head modeling and 3D cortical surface based source construction. The *FMRIB Software Library (FSL, version 5 or newer)* (Smith, 2002; Jenkinson et al., 2012) toolbox can be used to segment cortical surfaces from MRI images for the purpose of creating spherical conductor head models fit to individual brain anatomy (e.g., the inner skull surface). *BrainWave* also provides the option to use surface based source reconstruction by importing high-resolution cortical meshes from both the *CIVET* (Ad-Dab’bagh et al., 2005) and *FreeSurfer* (Fischl, 2012) software packages. In cases where FSL is not available (e.g., via Windows OS), a brain hull can be generated from these cortical surface meshes for the calculation of spherical head models, thus not requiring the use of FSL or SPM software.

While *BrainWave* has been tested extensively on all supported OS platforms and with various screen resolutions and versions of Matlab (**version 2013b is recommended**), we are aware that different OS/Matlab combinations can also introduce display issues, particularly font incompatibilities. Version compatibility and other platform support issues can be reported to the *BrainWave* support website which also provides an FAQ page for known issues.

### Data Organization, Installation, and Launch of *BrainWave*

**FIGURE 1:**
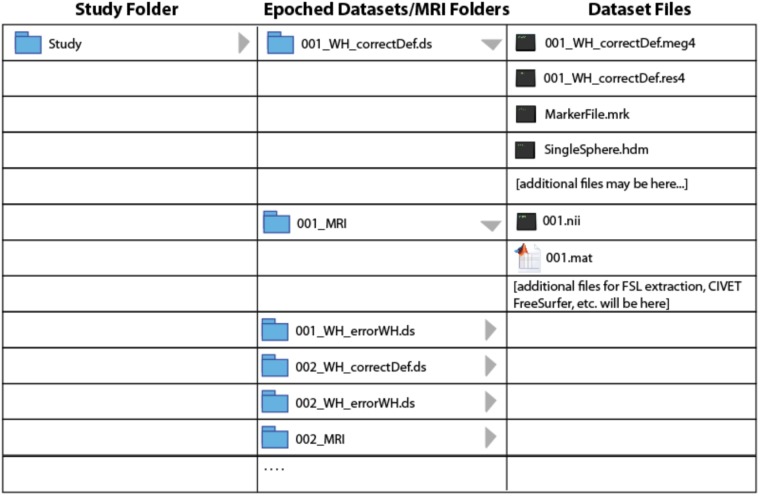
Data structure. MEG dataset and MRI directory file structure used by *BrainWave*.

*BrainWave* requires a fixed organization of MEG and MRI data files to minimize the need to search for files and to automatically link participant’s MEG and MRI data. Once imported and epoched, the organization of pre-processed study files is required to ensure seamless automation of certain *BrainWave* routines, such as group analysis where specific files are expected to reside within respective MRI or MEG dataset folders. In particular, all epoched datasets and converted MRI folders from all subjects must be saved within the **same** study folder hierarchy tier. **Figure 1** illustrates the **required** study folder hierarchy of condition-specific (^∗^.ds) datasets with respective subject-specific MRI folders. **Due to upload size limitations to figshare, we provide a customized Matlab script to simplify this setup by automatically unzipping and reorganizing the demo datasets into the required format seen in Figure 1**.

**FIGURE 2:**
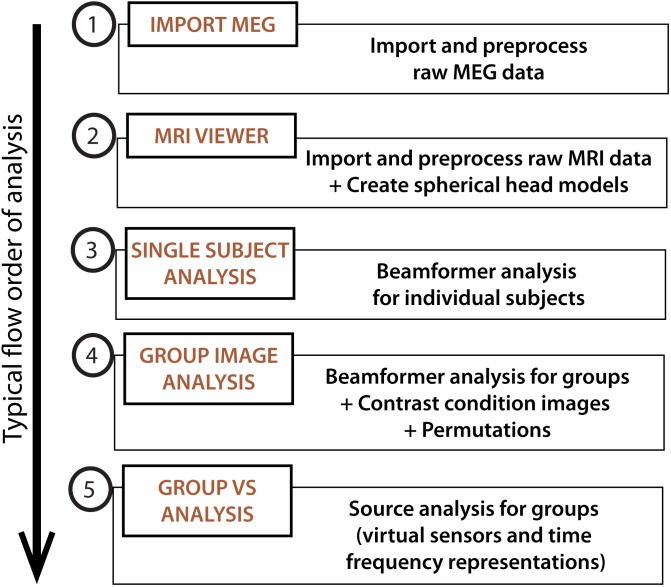
*BrainWave*. The main menu can be used to launch the main analysis modules in *BrainWave*, including (1) the import and preprocessing of raw MEG data, (2) MRI preparation for MEG co-registration, (3) single subject beamformer analysis for exploratory and/or single patient data analysis, (4) group beamformer analysis, and (5) an additional module for time course plotting and time-frequency decomposition from arbitrary or pre-selected brain locations.

• Download and install the latest version of the BrainWave toolbox (available at our University of Toronto website (cheynelab.utoronto.ca/brainwave). Download the demonstration package^4^, containing a customized file reorganization Matlab-script (*reorganize.m*), and 12 sets of MEG and MRI data folders. Unzip the package to a study folder (preferably located on a local drive). Twelve zip files should now be visible (subjectNumber_raw.zip).
• Open Matlab (version 7.5 or newer), and ensure the study folder is set as the current directory. Type ’*reorganize*’ into the Matlab Command Window to initiate the automated file unzip and reorganization script.
• For the spatial normalization to MRI coordinates and/or group analyses, you will also require a copy of the *Statistical Parametric Mapping (SPM8 and SPM12 currently supported)* (Friston et al., 2007) and *FSL* (version 5.0.0 or newer) for the calculation of head models. Download SPM software to the designated software folder, and install FSL as instructed on their website. In the Matlab Command Window, add the path locations to each program (*BrainWave*, SPM, and FSL). Note that additional environment set up for Matlab use may be required for FSL (see the FSL website for more information on how to do this), and is not compatible with Windows operating systems.
• To launch the *BrainWave Main Menu*, type ‘*brainwave’* into the Matlab Command Window. **Figure 2** shows the basic schematic layout of the *BrainWave* software in the typical order of processing steps as presented in this article.

### Preparing MEG Files

#### Import Raw MEG Data

Begin by ensuring that your demo datasets are unzipped, and that your study folder is the current Matlab directory. Note that preprocessing and epoching time, particularly when scanning for bad trials or head motion, will be significantly reduced if raw data files are stored on a local internal drive. When importing multiple datasets within the same study, the “batch” feature can be useful to avoid having to repeat all the same preprocessing steps many times over. Here, we will describe how to pre-process all raw datasets with the same parameters for two conditions as a single batch: (1) erroneous button presses to ‘withhold’ instructional cues and (2) correct default button presses. Note that *BrainWave* requires ‘raw’ data to consist of a single continuously recorded trial.

• Open *Import MEG* from the *BrainWave* main menu window.
• Enable batch processing by selecting *Open new batch*… from the *Batch* dropdown menu.
• As the demonstration data is already in CTF format, we select the raw (continuously recorded) datasets using the *File* →*Load CTF Datasets^5^* dropdown.
• Multi-select (hold-command key on MacOS or control key on Windows) all MEG datasets (folders ending in.ds) from the study folder.
• All successfully loaded datasets will appear in the *Dataset* dropdown list within the *Data Parameters* panel. A preview of the data, along with some details of the dataset collection, including acquisition parameters and fiducial coordinates, will also load to their respective fields.

#### Selecting Data Events and Epoch Parameters

• Each dataset has been recorded with unique and case sensitive marker names for each event^6^. Select *Use Event File* from the *Epoch Selection* panel, and click the *Load Event File* button.
• Open the *MarkerFile.mrk* file then select ***correctDef_4th*** from the event dropdown list which corresponds to the time of the button press for every 4th correct default (Go) trial. Since there are a very large number of default go trials relative to target trials (800 versus 200 trials, respectively), this event marker is provided to reduce computation time, and to make signal-to-noise levels more comparable across trial types. All event latencies will appear in the *Epoch Latencies* column list for the currently selected subject. Note that this list of latencies will update by selecting a different subject from the *Dataset* dropdown list.
• Input *Epoch Window* start value at -2 second (s) and end value at 2 s. Notice that the first latency in the *Epoch Latencies* column is invalid. This is due to the suggested epoch size exceeding the time prior to the first event (i.e., a trial will be rejected if the first event occurs at 1.5 s into an experiment, which does not meet the epoch start window requirement of 2 s). The next valid trial will be displayed in the *Preview* window.
• Bandpass filtering or powerline frequency notch filtering may also be applied at this time (*Filter Data* and *Filter powerline* in *Pre-processing* panel, respectively). The latter will remove the selected (50 or 60 Hz) powerline frequency and harmonics (notch width = 7 Hz). It is generally recommended to use minimal pre-filtering at this step as bandpass filtering will be applied to the epoched data during image generation. If *Use expanded filter window* option is selected the preprocessing filter will be applied to a time segment 50% greater than the epoch duration to avoid filter artifacts at trial boundaries. Powerline notch filtering is also optional, and not necessary for the demonstration data as this is CTF data with synthetic third-order gradient noise reduction applied.
• Once epoch parameters are selected, verify that you have a unique output file naming convention. *BrainWave* automatically creates dataset names based on the information provided within the raw dataset name (and header information, if present). The option to deselect checked auto-fill boxes is available as needed, as well as the option to change (or add) details using the *Subject ID* and *Label* boxes for a customized dataset name. *Recall that it is important that the subject ID appears first and prior to the first underscore*. Anything else may follow the underscore from the *Label* field, but it is highly recommended to use a simplified name describing the epoched event type or condition name, while ensuring that the created datasets will have unique filenames. For the example data, keep all default auto-fill options enabled (i.e., *Subject ID, Run ID*, and *Event Name)*. The final dataset name will appear in the *Save As* field as *^∗^_WH_correctDef_4th.ds*, where ^∗^ is the subject ID number.

#### Setting Channel Selection and Trial Rejection (Optional)

Epoched trials and channels can be edited manually, or automatically excluded via predefined thresholds. Manual editing of epochs may be done by scrolling through the epoch latencies in the *Epoch Latencies* list, pre-viewing the epochs in the *Preview* plot window and using the *Delete Events* button to remove the currently selected trial. Editing channels using the *Edit Channels* button opens a dialog that can be used to manually remove channels or subsets of channels with pre-defined “channel sets” selected from a drop-down menu (e.g., use only channels over left hemisphere, or by channel type e.g., use only gradiometer or only magnetometer channels). Custom channel set lists can be created to exclude noisy or malfunctioning channels. Note that the selected channel set will be applied to all datasets in any batch processing, and all datasets must contain the same number of MEG sensor channels. Datasets that contain a different number of MEG channels than the rest of the datasets in the current batch must therefore be epoched separately.

To avoid the time consuming and subjective process of manual data editing, *BrainWave* provides epoch rejection features that are automatically applied during single trial or batch epoching. The following sections describe the automated trial rejection features and how to apply them to the demonstration dataset.

##### Automatically exclude trials exceeding amplitude thresholds

Trials containing artifacts larger than a chosen amplitude threshold can be achieved by enabling ***Peak-to-Peak Amplitude Exceeds***… in the *Epoch Rejection* panel. For the demonstration data, conservatively set this value to a 3 picoTesla (pT). When using this option, it is necessary to enable the *Filter Data* option in the *Pre-Processing* panel and set the bandpass to, e.g., 1 to 100 Hz in the *Preview* panel to avoid rejecting trials with large DC drifts. This will exclude individual trials where the difference between the minimum and maximum peak amplitude exceeds three pT, or is equal to zero for the entire epoch (i.e., will also detect trials with flat channels).

##### Automatically exclude trials with resets

This feature works similarly to the amplitude threshold, with the exception that the threshold must be exceeded within one-time sample, resulting in only detecting amplitude steps (e.g., flux jumps). Since the CTF demonstration data contain no flux jumps this option can be left disabled.

##### Automatically exclude noisy channels

This can be used in combination with the amplitude threshold to exclude channels that cause an excessive number of trials to be rejected. In the demonstration data, Subject 07 contains several noisy channels that require this option to be selected. Click on ***Exclude channels where number of rejected trials exceeds…*** and set to a threshold of 90% (default). This will automatically add channels causing more than 90% of trials to be rejected to the “excluded” channel list and rescan the data. In non-batch mode, the excluded channels can be pre-viewed and edited, or saved as a custom channel set. Note that for Elekta/Neuromag data, this option can be used to automatically remove disabled channels that have been set to zero (flat channels).

##### Automatically remove trials based on excessive head motion (*CTF data only*)

If CTF CHL data are available, it is possible to exclude trials with excessive head motion by enabling the ***Mean sensor motion exceeds*…** option from the *Epoch Rejection* panel. For this example, we use the default 0.5 cm threshold. This will exclude any trial where mean MEG sensor motion (computed over all sensors or selected sensors if this option is selected) exceeds 0.5 cm. Motion is computed relative to the head, as defined by the fiducial coils position in device coordinates stored with the raw data, or the mean head position if this option has been selected. Information regarding the amount and range of sensor motion is displayed in the command window. This will noticeably increase processing time as it requires calculating the MEG sensor positions relative to the head at every time sample for all valid epochs.

##### Use mean head position (*CTF data only*)

If CTF CHL is available, the sensor geometry can be adjusted to reflect the mean head position for the epochs being analyzed by selecting ***Use mean head position*** from the *Pre-Processing* panel. For source localization, *BrainWave* defines the position and orientation of the MEG sensors (e.g., gradiometers) in a head-based frame of reference – identical to that used by the CTF software (i.e., the coordinate system defined by the three fiducial coil positions: nasion, left ear, and right ear). For raw CTF data, this is normally determined by the “head localization” measurements done at the beginning and end of each data acquisition, using by default, the mean of the two head positions. That is, even if CHL is enabled, the continuous head position data is not utilized. If CHL data is available, *BrainWave* will optionally allow you to use this data by averaging head position over only selected epochs during the epoching procedure. This provides a head position that reflects the actual head position for the data being analyzed, i.e., will exclude any large head movements between trials, or that may have occurred during the pre and post-head localization recordings. The adjusted sensor geometry (i.e., gradiometer position and orientations) are saved in the.res4 file and the mean fiducial locations (in dewar coordinates) are saved in the.hc file of the epoched dataset. The “Update” button can be used to preview the calculated mean fiducial locations (displayed in red font) for the currently selected dataset and parameters prior to epoching. Head position will be updated prior to epoching, and recalculated following trial rejection. If available, this option is recommended for an improved estimate of the true head position relative to the sensors, even if not rejecting trials for head motion. The updated overall head motion statistics after this adjustment are printed to the command window.

If you wish to immediately see the effects of the selected epoch rejection parameters, the *Scan Epochs* button can be used to preview which trials and channels will be removed for the currently selected dataset. After scanning, excluded trials or channels will appear as red in the *Preview* window and will be indicated with an asterisk (^∗^) in the channel and latency lists. This step can be repeated to determine the optimal parameters and thresholds prior to batch processing.

#### Adding New Conditions to Batch

The following is described to demonstrate the use of the batching process, but may be done in separate processes entirely.

• Click *Add to Batch* and select *Yes* from the pop-up window asking to add 12 datasets with current epoching parameters to batch.
• With the *Import MEG* dialog still open, click *Load Event File* button and choose *errorWH* from the *MarkerFile.mrk* file to epoch to a new condition in the same group of subjects. Select *Replace* from the pop-up to only epoch to *errorWH* event latencies.
• Click *Add to Batch* to include the *errorWH* condition to the epoching queue. Again, select *Yes* from the pop-up window asking to add 12 datasets to batch.

#### Close and Run Batch Mode

• The number of batch jobs should now appear in the *Batch* dropdown menu [“*Close Batch (2 jobs)*”]. Run both batch jobs by first selecting *Close Batch (2 jobs)*. Respond *Yes* when prompted whether to execute the batch process. The epoching process will start running during which time you will not be able to execute other Matlab commands. If you respond *No* you can run the batch process later by selecting *Run Batch*…. from the *Batch* dropdown menu. Note any non-executed batch settings will be lost if you close the group analysis window.
• You can monitor progress in the Matlab command window, which is also a useful source of information should an error occur. The entire process will take about one half hour or longer, depending on processor and hard drive/network speed (e.g., this should approximately take 12 min on a Macbook Pro with a 2.6 GHz i7 processor, 16 GB RAM and 256 GB SSD). If batch processing completes successfully, two epoched datasets will have been created for each subject: *^∗^_WH_correctDef_4th.ds* and *^∗^_WH_errorWH.ds*, where ^∗^ indicates each subject’s ID number.

### Preparing MRI Data

The preparation of MRI data includes the identification of fiducial placement required for the co-registration of MEG to MRI anatomy, as well as the need for spherical head models, which are used in the beamformer source localization calculation (see next section for more details). However, as mentioned in the introduction, the provided MRIs have undergone a de-identification process (i.e., a defacing tool that removes a large portion of the face). As such, much of the following preprocessing steps have already been carried out (including fiducial placement and the generation of FSL and CIVET cortical surface extractions) with the output files saved in the demonstration data package. Instead, this section will briefly discuss the generation of custom spherical head models using the provided pre-processed files generated with FSL (version 5.0.9). More options to create the necessary spherical models are available, particularly if FSL is not available (e.g., Windows users). See section III below or the user manual for more details.

#### Importing MRI Files

An anatomical MRI scan, typically a T1-weighted image, is required by *BrainWave* for the accurate co-registration of localized MEG activity to individual anatomy and subsequent template warping (using SPM) for group averaging. In the sample datasets provided, open the already converted DICOM to NIfTI file of each subject.

• Open *MRI Viewer* from the *BrainWave Main Menu* and under *File → Open MRI file…*, navigate to the MRI folder (e.g., *001_MRI*) and load the NIfTI file named *001.nii*.
• Use the brightness adjustment bar at the bottom of the window if the image appears too dark.

#### Fiducial Placement

• View each fiducial position using the *View Na*, *View LE* and *View RE* buttons.
• In the sample datasets, notice that such positions align to the three MEG head localization coil positions as marked to the center of each donut-shaped radiological marker^7^. Clicking anywhere within each view will move the orange crosshairs and subsequent position within the MRI volume. Saving new positions will update the fiducial position file (*001.mat*), but not necessary for this demonstration as the fiducials have already been optimally placed prior to de-identification. More details on how to set and save fiducials may be found in the user documentation.

#### Creating Customized Spherical Head Models Using FSL Surface Extractions

Single-sphere (and multi-sphere) head models are spherical conductor models of the head that are utilized in the simplification of the forward calculation of the brain’s magnetic field (Lalancette et al., 2011). To select optimal parameters (origin and radius) for these head models, the brain and three skull surfaces (inner skull, outer skull, and skin) are first extracted using FSL, if installed, from within the *MRIViewer* module. Alternatively, a convex hull describing the brain shape derived from third party software (e.g., CIVET and FreeSurfer) may also be used. Each surface type serves the purpose of optimally accounting for volume currents of a sphere, by fitting to the preferred inner skull surface (Hämäläinen et al., 1993), or aligned to the brain outer surface. The chosen surface will then be saved as the required surface shape (*^∗^.shape*) file for the head model calculation. Note that in MRIViewer ***shape*** data are in head or fiducial based coordinates (in cm), while ***surface*** data are in MRI voxel coordinates. The following steps perform the conversion between MRI voxel coordinates from the FSL.off file into CTF head coordinates and saved as the required CTF.shape format. However, other shape files can be loaded into *BrainWave* using the *Head Models* → *Load Shape file* menu option. In addition to shape files, this file options menu can be used to select KIT digitized head data (surface point files,.sfp), Polhemus data in a generic format (^∗^.pos files), or 3D data in GifTI format (.gii) for example to examine the accuracy of co-registration to digitized head surfaces, or use those surfaces to fit single or multi-sphere models. Here, we demonstrate the use of the FSL extracted inner skull surface (default option).

**FIGURE 3:**
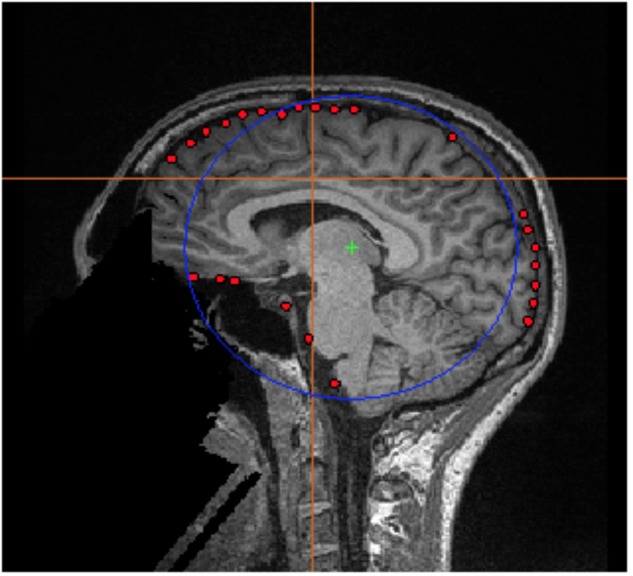
FSL Surface Extraction. Example of the inner skull surface extraction using FSL for subject 001 (red dots), with an overlaid single sphere head model (blue circle).

• With the current MRI loaded (*001.nii*), go to *Segmentation* → *Load FSL Surface*… and select *bet_inskull_mesh.off* to load the inner skull brain surface.
• Save this file as the required.*shape* file for the spherical head model calculation under *Segmentation → Save FSL surface as shape…* Name the file with an intuitive name (e.g., 001_innerSkull_FSL.shape).
• Finally, load the saved shape file (*001_innerSkull_FSL.shape*) under *Head Models → Load Shape File….*
• Go to the *Head Models* dropdown and select *Create Single Sphere Head Model*, then multi-select all epoched MEG datasets for that subject only. E.g., if 001.nii is loaded with the *001_innerSkull_FSL.shape* file, select *001_WH_correctDef_4th.ds* and *001_WH_errorWH.ds*. Note that multisphere head models depend on the relationship between the head position and sensors, so it is necessary to independently compute and save the models for each individual dataset. However, single-sphere models do not have this limitation and can be copied to other conditions using the *Copy Head Models* option in the *Group Analysis* dialog.
• Click *Save* to write a file called *singleSphere.hdm* to each dataset.
• A preview of the calculated sphere will appear (**Figure 3**).
• Repeat this Section “Creating Customized Spherical Head Models Using FSL Surface Extractions” for each subject. Ensure each epoched dataset per subject (e.g., *001_WH_correctDef_4th.ds* and *001_WH_errorWH.ds*) contains one file named *singleSphere.hdm*.

### Group Analysis

With datasets now prepared, the following sections will demonstrate the basic features of the toolbox. Section I describes how to setup the group analysis and conditions. Section II demonstrates group ERB analysis of time-locked (averaged) motor responses for two different conditions using common beamformer weights. Sections III to V demonstrate various options for viewing ERB images and time-courses and generate time-frequency plots. Sections VI and VII demonstrate the use of the SAM beamformer to image oscillatory motor activity and apply permutation thresholds. Finally, in section VIII, we demonstrate the ability to create a contrast SAM image between two conditions. For all examples, we provide recommended filtering and covariance parameters based on the initial analyses reported in Isabella et al. (2015). For a more detailed discussion of how to optimize parameter selection for beamformer methods, see (Brookes et al., 2008).

#### Prepare Study and Add Conditions

• Open the *Group Image Analysis* module from the *Main Menu*.
• Start a new study (*File* 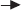 *New Study…*), and save it as *demo_STUDY.mat*.
• Go to *File* 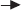 *Add Condition*… and select all epoched datasets labeled *^∗^_WH_correctDef_4th.ds*, where ^∗^ represents each of the subject ID’s. When prompted, name this condition *correctDefault*. Repeat to add *errorWH* datasets as a second condition. **It is recommended to save changes frequently (*File*** 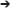 ***Save Study*) to avoid having to repeat these steps if you make an error**.

#### Generate Group ERB Images With Common Beamformer Weights

It is important to note that the number of trials in the default condition is significantly greater than those in the error condition, and that the low number of trials in some subjects for the error condition can possibly result in unstable matrix inversions (Brookes et al., 2008). To avoid differences between conditions that might be biased by differences in the computed beamformer weights, *BrainWave* provides an option to automatically combine datasets across conditions for a covariance computation that should provide a less biased comparison of source amplitude changes.

• To enable this option, under the *Condition 1* dropdown menu, select *correctDefault* from the condition’s list and *errorWH* in the *Condition 2* dropdown menu.
• Choose the option for beamformer weight computation labeled *Conditions 1 and 2 (common weights).* This will create a concatenated dataset of conditions 1 and 2 (e.g., *001_WH_correctDef_4th+001_WH_errorWH.ds*) to be used for the beamformer weight calculation.
• Select the *ERB:* radio button, and set Latency Range window size from -0.3 to 0.3 s with the default 0.005 s step size window. Press *Save*.
• Select *Volume (MNI Coordinates)* from the *Image Type* panel. Other image parameters such as *Imaging Volume* dimensions and voxel *Step Size* resolution (default is 4 mm) can be selected in the *Image Options* button pop-out dialog. Other options include using custom MRI templates for normalization, or applying a brain mask to the images (e.g., the inner_skull_mask.nii generated by FSL during creation of the head models). For most cases the default parameters are recommended.
• Finally, set the data bandpass and covariance parameters using the *Data Parameters* button. In this example, we have selected a time window and filter settings that are optimal for observing the transient brain responses (movement related fields) and associated frequency modulations that occur before and after movement onset as shown in previous studies (Cheyne et al., 2006). Set *High Pass* to 1 Hz and *Low Pass* to 30 Hz, change covariance window (ERB/VS Covariance) from -0.5 to 1 s, and load *singleSphere.hdm* into the custom head model. Note that a small amount of diagonal regularization (10 fT RMS) of the covariance matrix (applied prior to computation of beamformer weights) is set by default. This amount can be adjusted depending on the data. For robust data (large amounts of trials) no regularization may be necessary. Conversely, for data that may have been modified during denoising procedures such as ICA artifact removal or signal-space separation methods resulting in rank deficient covariance, regularization may need to be increased until a stable image is obtained.
• Go to *File* →*Save Study* to save all current parameters.
• Clicking *Generate Group Images* will prompt for a group analysis image name (in this case, an ERB for the condition listed in the *Condition 1* dropdown menu using the new combined datasets in the weight calculation). Choose a name for this group analysis on the condition selected under the *Condition 1* dropdown menu (example: *group_default_ERB*). A progress bar will appear and detailed messages indicating each step will be output to the Matlab command window. When processing is completed, the new combined datasets will be created which will be displayed in the group ERB image *4D Image Viewer*, normalized to the SPM template MRI (as specified in the *Image Options* dialog). This step will automatically generate combined datasets for common weight calculation, and run SPM to generate and apply the MNI template normalization parameters for each subject and may take several minutes or longer depending on processor speed.
• Finally, with the group analysis window still open, select *Condition 2* from the *Generate Images for*: panel to compute an ERB of the *errorWH* condition, using the same combined datasets in the common weight calculation. Keep all other parameters the same and click *Generate Images* to create a group ERB of the erroneous condition (recommended name: *group*_*errorWH_ERB*). Note that this analysis takes less time since we are using the same combined conditions and bounding box and both the covariance data and SPM normalization parameters have been computed in the previous step.
• If the image set window is accidentally closed, or you exit *BrainWave*, all previously created group analysis images can be quickly retrieved from the *Main Menu* (*File → Open ImageSet*…, then navigate to the group image folder and open the *^∗^_VOLUME_IMAGES.mat* file). Similarly, any previously generated group analysis, including all processing parameters, can be retrieved from the *Group Analysis* module *ImageSets* dropdown menu.

#### Navigating Images for Peak Source Activations

**FIGURE 4:**
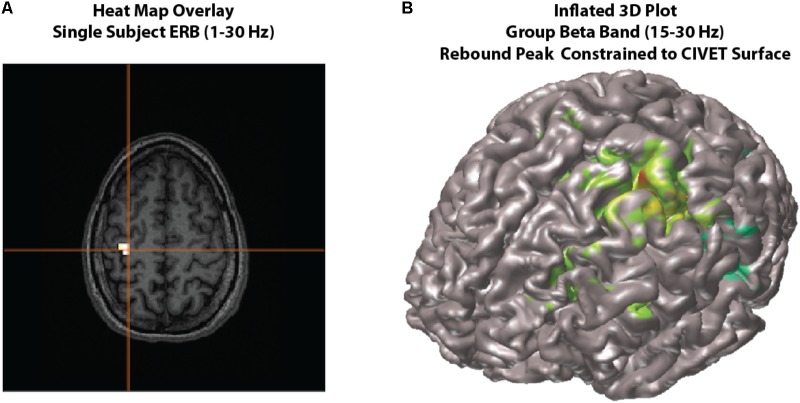
Viewing options. Examples of viewing options for source images. **(A)** Individual subject results can be overlaid onto their own MRI in the *MRIViewer* module. This example shows evoked activity (ERB) response of subject 002, overlaid onto their own MRI. Single subject or group images can also be viewed on a built-in template brain surface [FreeSurfer extracted pial surface from the Colin-27 (CH2.nii) average brain] or an averaged extracted pial surface from CIVET. **(B)** Shows an example synthetic aperture magnetometry (SAM) group analysis of a beta band (15–30 Hz) rebound peak, constrained to a CIVET extracted surface.

• Using the scrollbar at the bottom of each group beamformer glass brain 4D image, view peak information by selecting the *Show Peaks* (avoid setting threshold to less than 10% of the maximum value when using this feature as this will significantly slow updating of the list). Highlighting a peak from the list will display crosshairs at the respective position.
• In the present case, we are looking for a sensorimotor response where time zero is the button press. Keep scrolling until the peak reaches maximum strength of the motor field (**Figure 4A**). In the case of the correct default condition, magnitude strength will be located at a latency of approximately -50 ms (lower the threshold to ∼1.7 units), and the maximum peak location should read L Precentral Gyrus, BA 4 [Talairach Coordinates -38, -21, 54]. Repeat for the erroneous withhold condition where you will find peak latency at -70 ms (lower the threshold to ∼1.77 units) at L Precentral Gyrus, BA 4 [Talairach Coordinates -34, -25, 51].
• You can optionally view individual subject ERB images using the *Data* dropdown menu of the group image window.

#### Optional Viewing Features

In addition to the default glass brain images, source peaks may be presented in various ways. One way is to render the source localizations onto a rendered three-dimensional brain surface image, or as a “heat map” onto the individual’s own MRI (see **Figure 4**).

##### Heat map overlay

The traditional “heat map” of an individual’s result on one’s own MRI is also possible within *BrainWave* (**Figure 4A**).

• In the group ERB image, the *Data* dropdown menu will list the images of each subject analyzed for the current group image.
• For demonstration purposes, select the beamformer results for subject *001_WH_correctDef.ds* and navigate to the peak of interest at -50 ms. Under *File*, select *Overlay Image on MRI….* This will open the individual’s own MRI within the *BrainWave’s MRI Viewer*, and will overlay the current thresholded peak in “heat map” form onto the MRI.
• Adjust the threshold of the overlay using the *Overlay Threshold* scroll bar at the bottom of the window. The cross-hair cursor will automatically align to the largest peak.
• If needed, click *Find Peak* button (also located at the bottom of the window) to re-locate this position.

##### Plot 3D

• Within the group image for the *correctDef* condition, click the button labeled *Plot 3D* in the top right corner of the image window. This will generate a 3D rendered image (using 3D linear interpolation) of the group beamformer source volume of the current latency onto the *Freesurfer* extracted brain surface of the Colin-27 (CH2.nii) average brain^8^.
• One may also generate beamformer images constrained to each individual subject’s cortical surface with sources placed at each vertex of the cortical mesh, with the option to constrain source orientation to be normal to the cortical surface (**Figure 4B**). To demonstrate this option, high-resolution CIVET surface extractions have been provided for all subjects.
• To utilize surfaces for surface constrained beamformer analyses, change the *Image Type* by selecting *Surface* instead of *Volume* within the group (or single subject) analysis window.
• Click *Select*, then choose from the CIVET files provided (e.g., CIVET_pial_SURFACE.mat). These files contain pre-calculated whole brain meshes co-registered to the subject’s MRI volume and the MEG head coordinates, along with associated data needed for surface imaging all saved in a ^∗^_SURFACE.mat file for each subject. Step by step instructions on the generation of these MAT files are provided in the *BrainWave* documentation and not repeated here.
• You may optionally preview the surface by using the *View* dialog.
• Set up your ERB or SAM analyses as usual. Select the *Use surface normal constraints* checkbox in *Image Options* if you wish to constrain the source orientation to be normal to the surface at each vertex. This option assumes co-registration errors are small. If not selected, the optimal orientation at each vertex is computed similarly to the scalar beamformer option for volumetric images.
• Press *Save*, then click *Generate Images* to create a 3D plot rendering of all subject results (see **Figure 4B** for example beta rebound SAM result).
• Various options for displaying inflated surfaces, rotating and peak finding within the 3D window are described in detail within the *BrainWave* user’s manual.

#### Generate Frequency Specific Time Course Plots From Peak Sources

*BrainWave* has been integrated with two options that illustrate various frequency-specific time-course plots for the selected source peaks. The *BrainWave* dropdown menu in each contains tools to adjust parameters specific to the calculated result, and are described in detail within the user documentation. All other editing options (e.g., line thickness, overlaying plots, adding legends, etc.) may be found under standard Matlab menu capabilities.

##### Virtual sensor (VS)

Compute peak source activity time courses of specific frequency ranges with a VS plot.

**FIGURE 5:**
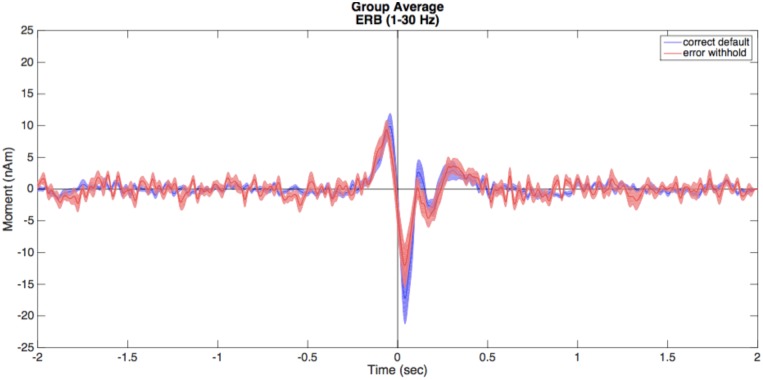
Virtual sensors (VS). Illustrated here are the averaged VS plots calculated from the ERB peak of each condition. Correct default (in blue) and error withhold (in red) plots are displayed with shaded standard error bars using *BrainWave* tools, then edited to a single plot using Matlab’s figure editing tools (e.g., overlay, add legend, text size, etc.).

• In the beamformer image, highlight a peak from the peak list and click *Plot Group VS*. The selected peak location will be converted to MEG head coordinates for each subject (unwarped) prior to computing the time series.
• Choose *Find largest peak within 10 mm search radius* and click *OK*. This option attempts to use the closest peak location in each individual subject’s source image rather than the group mean, generating more accurate amplitude measures.
• In this window, we can select our frequency range independent of what was calculated for the beamformer image using the *Data Parameters* pop-up. For now, we will keep these settings the same and close the *Data Parameters* window.
• As some individuals may show varying dipole polarity differences, we are able to force all polarities into a single direction. In these example data, we have a known evoked motor field peak around the -50 ms time point for the *correctDefault* condition. Enable the *Make Polarity Positive* options under the *Virtual Sensor Plot* panel on the left, then enter -0.05 to force all peaks at this time point to display in the positive direction.
• Click the *Plot VS* button to generate a group averaged time course.
• Repeat this for both conditions (correct default and error withhold) where time zero is the button press. A combination of options in the *BrainWave* menu and standard Matlab editing tools (e.g., copy and paste) can be used to further customize figures, such as that shown in **Figure 5** overlaying the VS time courses and their standard error for two conditions.

##### Time-frequency representation (TFR)

It is also possible to view the VS time-series data as a time-frequency representation plot, or TFR. This is useful for guiding the time-frequency analysis, and selecting time windows for SAM beamformers (as shown in the example shown).

**FIGURE 6:**
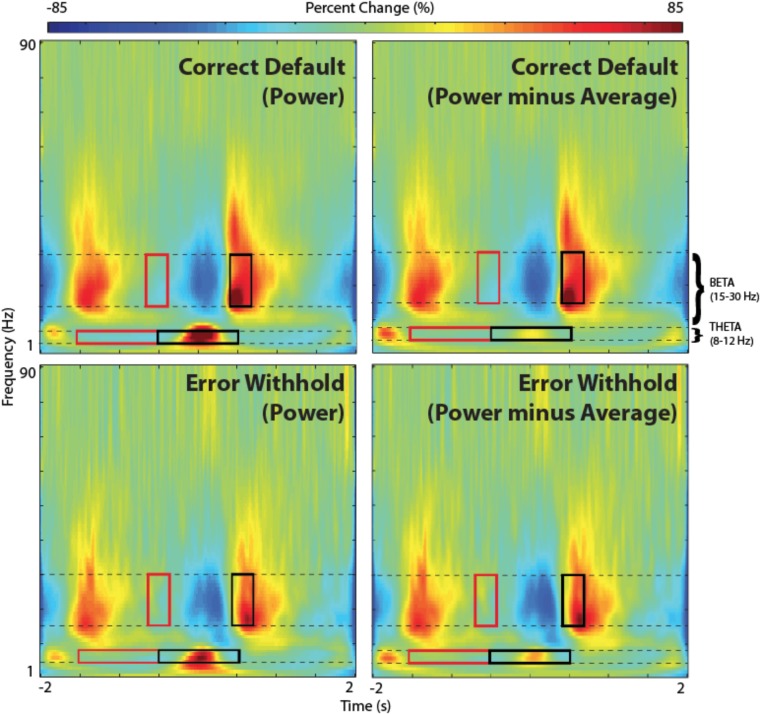
Time-frequency representations (TFR). TFR plots are useful as an aid in the identification of appropriate baseline and active windows for SAM beamformer analyses. Here, we demonstrate the chosen baseline window (red) and active window (black) within the beta band frequency (15–30 Hz), as well as suggested windows for theta band frequency (4–8 Hz), for a group SAM motor peak analyses. Time zero indicates button press. Note that removing the averaged evoked activity (power-average dropdown option within *BrainWave*) shows only non-phase locked activity, resulting in reduced power in both beta and theta bands. However, larger reductions are found in theta and remains higher in the error condition.

• In the beamformer plot, highlight the peak again within the plot list and click *Plot Group VS* then select *Find largest peak within 10 mm search radius* and click *OK*.
• In this window, open *Data Parameters* and set the frequency range from 1 to 90 Hz. Click Save to exit the *Data Parameters* window.
• In the *Time-Frequency Plot* panel on the bottom left, select the type of time-frequency transformation and parameters to be used. In this case we will use default settings: select Morlet wavelet transformation (recommended) with 1 Hz frequency bin size and a time-frequency resolution (approximate number of cycles per wavelet) of 7 [For details on the Morlet wavelet algorithm and choice of wavelet number see (Tallon-Baudry and Bertrand, 1999)].
• Click on the *Plot TFR* button.
• Repeat this for both conditions (correct default and error withhold) where time zero is the button press.
• Note, phase-locked evoked responses can be removed from the TFR by selecting *Power-Average* from the *BrainWave* 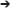 *Plot* dropdown option (**Figure 6**).

#### Generate Group SAM Beamformer (Beta Band – 15–30 Hz)

Determining optimal time and frequency windows of baseline and peak activity/rebound responses can be done using the TFR generated from the ERB image (**Figure 4A**) in guiding active and baseline time window selections (see **Figure 6**).

**FIGURE 7:**
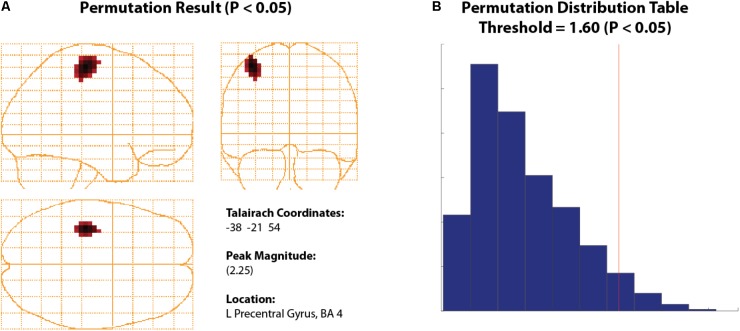
Permuted Beta Suppression Peak. **(A)** Significant beta rebound peak with all voxels shown with a significance of *P* > 0.05 or higher. **(B)** The significance cut-off was determined using a permutation distribution plot which calculated all significant values to the right of the red vertical line.

• Open the group study window (*Group Image Analysis* button from the *Main Menu*) load the study (*demo_STUDY.mat*) under *File* 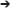 *Open Study*.
• Set your *Data Parameters* to view beta bandpass with a high pass of 15 Hz to a low pass of 30 Hz, and ensure that *singleSphere.hdm* is selected.
• Note that in this case, the covariance window only applies to computation of VSs as the SAM algorithm uses the baseline and active windows periods only to compute the weights for the pseudo-T images. Thus, we make no changes to the covariance window.
• Choose the *correctDef* condition from the *Condition 1* dropdown menu, *errorWH* in the *Condition 2* dropdown menu, and select the customized beamformer weight computation called *Conditions 1 and 2 (common weights)*, and *Condition* 1 from the *Generate Images for* option to create a group image for correct default condition, using common weights between conditions.
• Select the *Synthetic Aperture Magnetometery* (or *SAM*) radio button, *Pseudo-T* and set the baseline window to -0.7 s (start) and -0.4 s (end), then set the active window from 0.4 to 0.7 s. Sliding windows are ideal in searching for peaks. However, for demonstration purposes, we describe the generation of a SAM image for the rebound peak (based on the beta windows found in **Figure 6** TFR). Refer to the user documentation for more information on how to perform sliding window analyses.
• Click *Generate Group Images* then save the group name as *correctDef_beta_SAM* when prompted. After several minutes, this will create a new glass brain beamformer within the beta band range (e.g., **Figure 7A**).
• Unlike ERB images, SAM images are not rectified and can be displayed as positive or negative values (increases or decreases in power relative to baseline in the pseudo-T and pseudo-F images, respectively). Since the glass brain display can only show one polarity at a time, the Negative image data can be viewed using the *Show Negative* radio button.
• In this example, to find the (positive) beta rebound peak, ensure the *Show Positive* radio button is enabled. Peak beta rebound location should be -30, -17, 51 L Precentral Gyrus, BA 4. In the next section, we will run a statistical permutation to determine the significance of this peak.
• Repeat these same steps for error withhold, using the same active and baseline windows for comparable results between error withhold and correct data.

#### Thresholding Using Permutation Tests

To statistically threshold source images, *BrainWave* includes a simple non-parametric permutation test (Nichols and Holmes, 2002; Chau et al., 2004; Lin et al., 2006) that can be applied to group volumetric images (with ROI options) or surface images that contain both positive and negative values (e.g., SAM pseudo-T images or any contrast image).

• To view an example of this option, navigate to the beta rebound image from the previous analysis for the 0.4 to 0.7 s active window. Under the *Data* dropdown menu, click *Permute Images* to open the Permutation Test parameter selection.
• Set *Alpha* to 0.05, select *Corrected (omnibus)* and *Use ROI Xmin = -75, Xmax = 75, Ymin = -112, Ymax = 75, Zmin = -50, Zmax = 85*. The *Number of Permutations* is set automatically based on the number of subjects provided. For 12 subjects, this number should read 2048 – the maximum number of iterations based on 2^N^, where N is the number of subjects. In *BrainWave*, the number of iterations has been optimized for about 11 subjects, with limitations on groups less than 8 (Chau et al., 2004). Optionally, you may select *Plot Distribution* to view the resulting permutation distribution histogram.
• Click *Run*.
• The resulting image will show all significant peaks (**Figure 7A**) at and above the significance threshold as indicated by the red vertical significance ‘cut-off’ line in the permutation distribution plot (**Figure 7B**). In the provided data, the beta rebound peak of interest appears as most significant. Selecting the confirmed significant peak from the *Show Peaks* window is now available for further waveform analyses as described earlier (e.g., VS or TFR). For more details see (Chau et al., 2004).

#### Generate Group Contrast Beamformer (Theta Band – 4–8 Hz)

Contrast images are also possible in *BrainWave*. Here, we will demonstrate the analysis of increased frontal theta (4–8 Hz) oscillations on error trials by creating an error minus correct (error > default) contrast image, with the use of common weights and creating a *Pseudo-T* SAM image subtraction.

**FIGURE 8:**
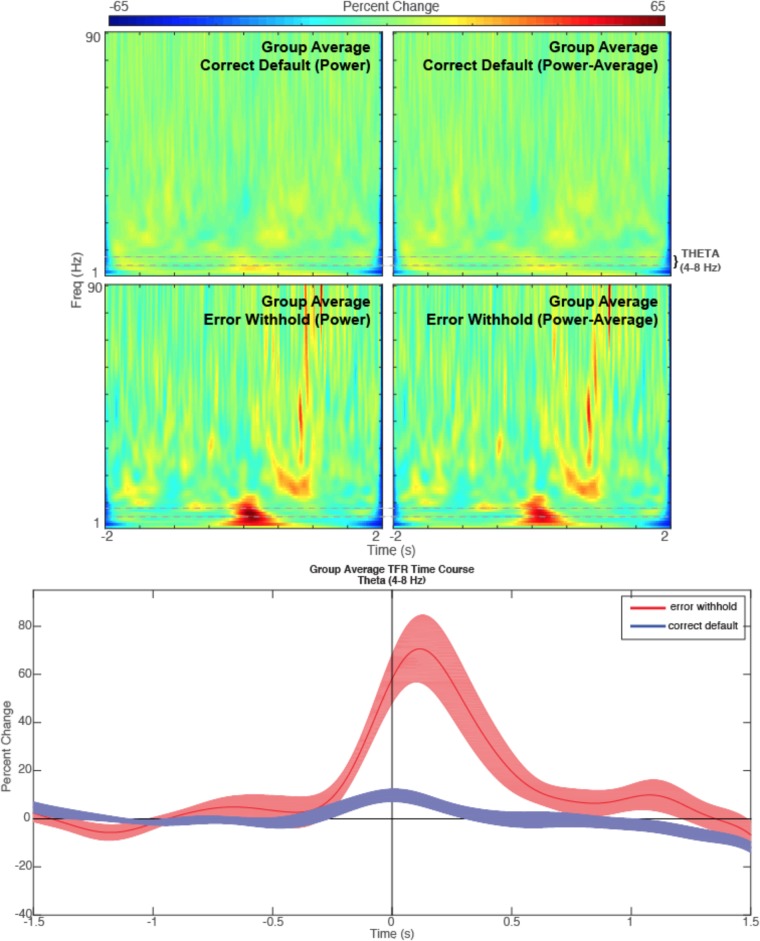
Theta. Time-frequency representations are shown for the R Anterior Cingulate peak (top) generated from a contrasted (error withhold > correct default) group theta band (4–8 Hz) beamformer as described. Note that the theta band power remains present in error peaks when phase-locked activity is removed (power minus average). A time course of the theta band TFR is represented (bottom) with shaded error bars for each condition described.

• In the *Group Image Analysis* window, open the *Data Parameters* window. Set bandpass to a low pass of 8 Hz and high pass of 4 Hz; covariance window to -2 to 2 s (to capture low frequency activity), and keep the same head model as above (*singleSphere.hdm*). Click *Save* to close *Data Parameters*.
• In the *Beamformer Parameters* panel of the group image analysis window, set baseline from -1.5 to -0.5 s, and change the active window to -0.5 to 0.5 s.
• Create a contrasted image by first setting *Condition 1* dropdown to *errorWH* and *Condition 2* dropdown to *correctDef*, then enable *Condition 1 minus Condition 2* and select *Conditions 1 and 2 (common weights)* from the *Compute beamformer weights using:* panel.
• Click *Generate Images* to run the test. Input a save name, e.g., theta_err-def.
• The resulting image should show strong theta peaks in frontal brain regions (left medial frontal gyrus and right anterior cingulate).
• To see VS timecourse differences of theta activation between both contrasted conditions, select the *R Anterior Cingulate* from the 4D image peak list, and click *Plot Group VS*.
• For contrast images, VS and TFR plots will be automatically generated for both conditions unless otherwise selected from the dropdown list within the *Time-Frequency Plot* panel of the *Virtual Sensor Analysis* window. Ensure this is set to *Plot Conditions 1 and 2* to generate both TFR plots for comparison. Set *Data Parameter* bandpass to 1 Hz high pass and 90 Hz low pass. Click *Plot TFR*.
• In each TFR plot, change the plot mode to *Power-Average (*power minus average*)* using *BrainWave* 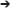 *Plot* dropdown menu to remove phase-locked evoked signal from the TFR. This will remove any low frequency power that might reflect the power in the evoked response rather than induced (non-phase locked) theta oscillations. The resulting TFR plots should appear similar to those as shown in **Figure 6**.
• To view only theta oscillations in the form of a narrow-band time course plot, repeat the above steps to select a peak and generate a TFR, but change the low pass to 8 Hz and high pass to 4 Hz.
• Remove phase-locked signal (*Power-Average*), then select *BrainWave* 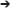 *Show Timecourse*.
• We can correct for signal averaging edge effects by setting the same baseline for each condition. To do this, go to *Brainwave* 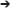 *Plot Parameters*, then save *Baseline* as -1.5 s start time and 0 s end time in each figure.
• Finally, set error bars (the standard error of all subjects at each time interval chosen) and color options from the *BrainWave* dropdown menu prior to overlaying plots using in-built Matlab figure tools.
• The resulting image (after manually setting identical colorbar ranges for each plot using Matlab tools) is found in **Figure 8**. Note that the *errorWH* condition shows a large induced theta burst around time zero, while *correctDef* TFR shows little or no increase in theta activity at the same time.

## Discussion

*BrainWave* offers a simple graphical interface Matlab toolbox for performing minimum-variance beamforming analysis of MEG data, with rapid and interactive visualization of evoked and induced brain activity. As demonstrated here, the latest implementation includes a group analysis module with automated processing steps to allow the computation and display of group averaged source images and time courses with minimal user intervention. The GUI interface simplifies setup time and eliminates the need to write customized Matlab code, yet can rapidly generate a four-dimensional source image dataset in several seconds through the use of compiled library routines on most notebook or inexpensive desktop computers.

Default options are provided to allow the rapid generation of source images and waveforms for exploratory analyses, while maintaining flexibility in the choice of preprocessing and modeling parameters. For example, we demonstrated here the ability to utilize data covariance from multiple conditions (“common weights”) to reduce spurious differences due to variations in the beamformer weights, as well as increased stability of the covariance matrix inverse for weight calculation (Brookes et al., 2008).

For brevity, only the basic steps to proceed from raw data to a group analysis using a simple Go/No-go task are described. More detailed tutorials with additional background information and examples of customizable options not covered in this tutorial, can be found in the software user’s documentation. Additional content includes the ability to use “surrogate” MRIs [using adult or child MRI templates (Holmes et al., 1998; Fonov et al., 2010)] if MRI data is not available; options for creating and viewing source time courses, including difference waveforms, and the ability to export source images and time courses to other software platforms for additional statistical analyses.

Although the current tutorial focused on group analysis, *BrainWave* also includes a separate module for single subject analysis. This is particularly useful for clinical applications where group averages are not performed. For example, importing externally selected epileptiform spike latencies to compute event-related ERB images around only those time points (Mohamed et al., 2013). These images can then be evaluated outside the toolbox for averaging or individual inspection. All image generation and display options described for group analyses are available in the single subject module, in addition to the ability to view source images in the non-normalized CTF coordinate system.

Human neuroimaging with MEG increasingly relies on the combination of structural and functional images to study the time-resolved activation of neural circuits distributed throughout the brain. This requires the integration of many different, and often complex, computational techniques to collect, organize and integrate large amounts of functional and anatomical data. Accordingly, the intended purpose of the *BrainWave* toolbox is not to provide an exhaustive library of signal processing and source reconstruction algorithms, but to integrate commonly used approaches to beamformer source analysis into a single platform for analysis and visualization. An important design philosophy for *BrainWave* is to provide a fast and interactive platform that is ideal for exploratory analysis of MEG data. This provides immediate visualization of localized sources and their time courses, with built-in peak finding and anatomical labeling, rather than a ‘black-box’ pipeline that requires the use of anatomical templates and separate tools or script building for data visualization.

*BrainWave* has been used in a number of published studies from our lab and others (Mohamed et al., 2013; Cheyne et al., 2014; Dockstader et al., 2014; Mersov et al., 2016; Pu et al., 2017) and has a growing user base. Enhancements and additions to the *BrainWave* toolbox with semi-annual releases are communicated to our user base and announced on our website. Future additions will include, but are not limited to: support for additional import and export file formats including new MEG vendor formats, as well as future standardized MEG data formats such as MEG-BIDS (Niso et al., 2018); improved methods for MEG-MRI co-registration and fiducial placement; an integrated equivalent current dipole fitting module; the implementation of PCA/ICA methods for extracting patterns of source activity; and additional statistical tests for group images and time-frequency analyses [e.g., waveform comparison analyses for multiple conditions (Guthrie and Buchwald, 1991)]. Other planned additions include more streamlined integration of the output of *BrainWave* with other analysis toolboxes that can perform secondary analyses, for example correlational or connectivity analysis (Granger causality, phase-amplitude coupling) between source time courses. We believe this approach will avoid unnecessary duplication of methods, and improve standardization of MEG analysis techniques based on beamformer source reconstruction.

Currently, *BrainWave* does require a dedicated processing machine with high demands for memory and graphics resources and is not suited for running parallel processes. Although the current version of *BrainWave* is not directly scriptable, Linux and Mac OS compatible command-line programs that can perform some of the core *BrainWave* functions are available on our website^9^ and can be used to build stand-alone scripts to generate images and waveforms. These are based on the same C++ library subroutines and the resulting output files are compatible with *BrainWave* with future potential for generating more efficient pipelines for large group analyses, while maintaining the visualization options available in the Matlab toolbox.

## Author Contributions

CJ, DC, and PF contributed to the on-going development of the *BrainWave* software and to the writing of manuscript. Example MEG and MRI data was provided by SI.

## Conflict of Interest Statement

The authors declare that the research was conducted in the absence of any commercial or financial relationships that could be construed as a potential conflict of interest.

## Footnotes

1 The *fiff2ctf* conversion program compilation (required for converting *Elekta/Neuromag* datasets into CTF format) is currently available for Linux distributions only. A Macintosh version is planned for future release.

2 *KIT/Yokogawa* users must first export their data using the BESA export feature.

3 http://talairach.org

4 https://figshare.com/s/2e1c6559cadae29429bc

5 Data from other manufacturers (e.g., .*fif* or *.con* files) must be imported using, *File* → *Import MEG data…*, which then converts each to the CTF *.ds* format in a single step.

6 Event marker labels: ***NewLightFiltered***, the filtered photodiode light trigger of each visually presented stimulus; ***rightIndex***, indicates a right index finger button press; ***nonTarget***, event label of a non-target trial; ***Target***, event label of a target trial (i.e., the number 3); ***errorWH***, an incorrect withhold trial time-locked to the button press; ***correctDef***, a correct non-target trial time-locked to the button press*;* ***correctDef_4th***, every fourth trial of *correctDef*.

7 Fiducials are the identified positions of each MEG head localization coil. In this experiment, the nasion (*Na*) will roughly align to the bridge of the nose at the level of the middle of the eye, while the left (*LE*) ear and right (*RE*) ear positions will align to just anterior to the tragus of the ear.

8 http://www.bic.mni.mcgill.ca/ServicesAtlases/Colin27

9 http://cheynelab.utoronto.ca/commands
